# Correlation between hepatic steatosis severity diagnosed by ultrasound and metabolic indexes in elderly patients with MAFLD

**DOI:** 10.3389/fmed.2024.1467773

**Published:** 2025-01-07

**Authors:** Zhitang Liang, Renhao Huang, Lingyun Zhang

**Affiliations:** General Practice Department, Binzhou Medical University, Yantai, Shandong, China

**Keywords:** liver fat content, ultrasonography, metabolic-associated fatty disease, elderly, metabolic parameters

## Abstract

**Objective:**

To explore the connection between metabolic parameters and the severity of hepatic steatosis determined through ultrasound in elderly individuals with metabolic dysfunction-associated fatty liver disease (MAFLD).

**Methods:**

4,663 senior individuals who were 65 years of age or older were included in this research. They were examined physically at the Ninghai Street Community Health Service Center in Yantai City between June 7, 2021, and October 15, 2021. There were two categories of individuals identified: the MAFLD group (*n* = 2,985) and the non-MAFLD group (*n* = 1,678). Based on liver ultrasonography results, individuals in the MAFLD group were further separated into three groups: mild (*n* = 2,104), moderate (*n* = 766), and severe (*n* = 115). To identify indicators of risk for the severity of hepatic steatosis, metabolic data was contrasted between the groups employing logistic regression.

**Results:**

In comparison to the non-MAFLD group, the MAFLD group showed significantly elevated levels of body mass index (BMI), blood pressure, gender, age, lipid profile, alanine transaminase (ALT), and fasting blood glucose (FBG; *p* < 0.05). Among individuals with MAFLD, there was a positive correlation between BMI, FBG, ALT, and aspartate transaminase (AST) levels and the severity of hepatic steatosis (*p* < 0.05). Logistic regression analysis indicated that BMI, female gender, FBG, ALT, triglycerides (TG), and serum uric acid (SUA) constituted risk factors for increased severity of hepatic steatosis in MAFLD.

**Conclusion:**

The severity of hepatic steatosis in elderly MAFLD patients is significantly correlated with female gender, BMI, ALT, FBG, TG, and SUA.

## 1 Introduction

Metabolic dysfunction-associated fatty liver disease (MAFLD), originally known as non-alcoholic fatty liver disease (NAFLD) (1), has practical and straightforward diagnostic criteria that are superior to NAFLD for determining individuals at elevated risk for liver fibrosis and extrahepatic manifestations, including chronic kidney disease (CKD), type 2 diabetes mellitus (T2DM), and cardiovascular disease (CVD) (2). Currently, its global prevalence has sharply risen to 25% (1), with a prevalence rate as high as 32.3% in China (3). On a worldwide scale MAFLD is an extremely prevalent type of chronic liver disease (4), and among the top causes for mortality from intrahepatic complications (5). The hazards of MAFLD not only include its propensity to cause liver fibrosis and hepatocellular carcinoma but also its association with extrahepatic complications, such as cardiovascular and cerebrovascular diseases, CKD, T2DM, and related malignant tumors, posing significant health risks that cannot be overlooked (6).

However, with the rapid aging population in Asia (7), studies have shown that aging, male gender, and the presence of menopause is a major risk factor for MAFLD (8). A meta-analysis of global MAFLD prevalence has indicated a continuous increase in prevalence across all age groups from 30–39 years to 70–79 years (9). Additionally, individuals aged ≥50 years are more susceptible to MAFLD progression in comparison to younger populations (10). MAFLD typically progresses to advanced stages before manifesting significant symptoms (11). Moreover, given that MAFLD can shorten life expectancy by up to 4 years (12), the need for early detection and screening becomes increasingly important (13).

Liver biopsy are thought to be the most accurate way to diagnose fatty liver disease, however, its invasive nature, risk of bleeding and infection, as well as high costs render it impractical for large-scale screening (14). For determining the presence of fatty liver disease, ultrasound is the most effective and frequently employed technique (15). Moreover, research has demonstrated that ultrasound exhibits an approximate 60% diagnosis accuracy for mild liver steatosis, whereas its accuracy in diagnosing moderate and severe hepatic steatosis is around 90 and 95%, indicating great accuracy in detecting the severity of hepatic fat accumulation (16). Liver steatosis can be detected with excellent specificity and sensitivity using MRI, particularly MRI-PDFF. All grades of steatosis in MAFLD patients can be detected more accurately with MRI-PDFF than with ultrasound because it allows fat mapping of the entire liver (AUROC 0.99) (17). However, MRI-PDFF, typically employed as a research tool, is unlikely to be applied as a first-line screening method in clinical practice due to its logistical complexities, lengthy scan time, and the lack of required expertise at most medical imaging centers.

One important risk factor for metabolic diseases is obesity.Adipocytes in obese people become hypertrophic and dysfunctional, which changes the production of adipokines like adiponectin and leptin.Systemic insulin resistance is exacerbated by adipose tissue malfunction, which fosters ectopic lipid buildup and persistent low-grade inflammation (18).Adipose tissue free fatty acids (FFAs) are released more often in insulin-resistant individuals, and their livers absorb more of them. These FFAs are subsequently converted to hepatic triglycerides, which causes hepatic steatosis (19).However, FFAs also cause hepatic and peripheral insulin resistance, and insulin resistance feeds the vicious cycle by causing more hepatic FFAs to build up (20).It is noteworthy to emphasize that interactions between genes and the environment appear to be essential for hepatic steatosis (20).For instance, there is a high correlation between variations in liver fat content and variations in the patatin-like phospholipase domain-containing 3 (PNPLA3) gene. PNPLA3 is a protein that is expressed in hepatocytes and adipocytes. It is an acyl hydrolase that hydrolyzes triacyl-, diacyl-, and monoacylglycerol (21). Insulin resistance and the buildup of liver fat are linked to the rs738409 polymorphism, which is linked to the loss of the hydrolyzing activity of proteins (22).Moreover, numerous studies have shown that a Mediterranean diet is related with a lower incidence of hepatic steatosis, while a high-fat Western diet is connected to an increased risk of the condition (23).

Referring to previous literature (24, 25), we selected age, gender, BMI, fasting blood glucose (FBG), and blood pressure as potential risk factors based on their critical roles in the pathophysiology of metabolic-associated diseases and fatty liver. Age is a major risk factor for MAFLD, with studies showing that advancing age is associated with significant changes in fat distribution, insulin sensitivity, and metabolic functions, which may increase the risk of hepatic fat accumulation. Older adults, in particular, experience a decline in hepatic insulin sensitivity and associated metabolic disorders, making them more prone to severe fatty liver. Gender also plays an important role in the development of MAFLD, with gender differences potentially linked to hormonal changes. Postmenopausal females, due to declining estrogen levels, may experience reduced hepatic fatty acid oxidation and increased fat accumulation. Additionally, the increased visceral fat ratio in postmenopausal women is thought to contribute to the progression of fatty liver disease. BMI is a core risk factor for MAFLD. Obese individuals, especially those with visceral fat accumulation, are more likely to develop hepatic fat deposition. Studies have shown that for every unit increase in BMI, the risk of MAFLD significantly increases. Furthermore, obesity leads to insulin resistance and chronic low-grade inflammation, further exacerbating hepatic fat accumulation. FBG reflects the state of glucose metabolism, and its elevation is often associated with insulin resistance, a key pathological mechanism of MAFLD. Blood pressure is closely linked to metabolic syndrome. In hypertensive patients, arterial stiffness and endothelial dysfunction may affect hepatic blood supply, further triggering hepatic metabolic abnormalities. Elevated blood pressure may also share common mechanisms with insulin resistance and hepatic fat accumulation (26, 27).

Nevertheless, there is currently insufficient research investigating the connection between metabolic disturbances and hepatic steatosis severity in elderly MAFLD populations. Exploring the connection between metabolic parameters and the severity of hepatic steatosis determined via ultrasonography in elderly MAFLD individuals is the purpose of this research. The objective is to provide a basis for risk stratification of elderly MAFLD populations at the grassroots level and to address the necessity of referral to higher-level hospitals for MAFLD diagnosis and management.

## 2 Materials and methods

### 2.1 Study design

A cross-sectional investigation was carried out utilizing the medical records of individuals who had physical examinations at the Ninghai Street Community Health Service Center in Muping District, Yantai City, from June 7, 2021, to October 15, 2021. A total of 4,663 elderly individuals aged ≥65 years were enlisted as participants in the research. Depending on whether MAFLD was present or not, individuals were separated into two groups: the MAFLD group and the non-MAFLD group. The basic characteristics and laboratory parameters of the two groups were compared. The MAFLD group was separated into three subgroups based on the severity of hepatic steatosis: mild, moderate, and severe. The basic characteristics and laboratory parameters among these sub-groups were also compared. Both univariate and multivariate analyses were applied to delineate independent risk variables.

### 2.2 Data collection

Every registered participant had a physical examination, during which their gender, age, diastolic and systolic blood pressures (DBP and SBP) were measured. The body mass index (BMI) was calculated utilizing the height and weight values. The community health service center’s laboratory department performed various blood tests, such as triglycerides (TG), high-density lipoprotein cholesterol (HDL-C), alanine transaminase (ALT), fasting blood glucose (FBG), and serum uric acid (SUA) et al. Additionally, all participants underwent abdominal ultrasound examinations to evaluate hepatic steatosis.

### 2.3 Diagnostic criteria

**Figure 1:**
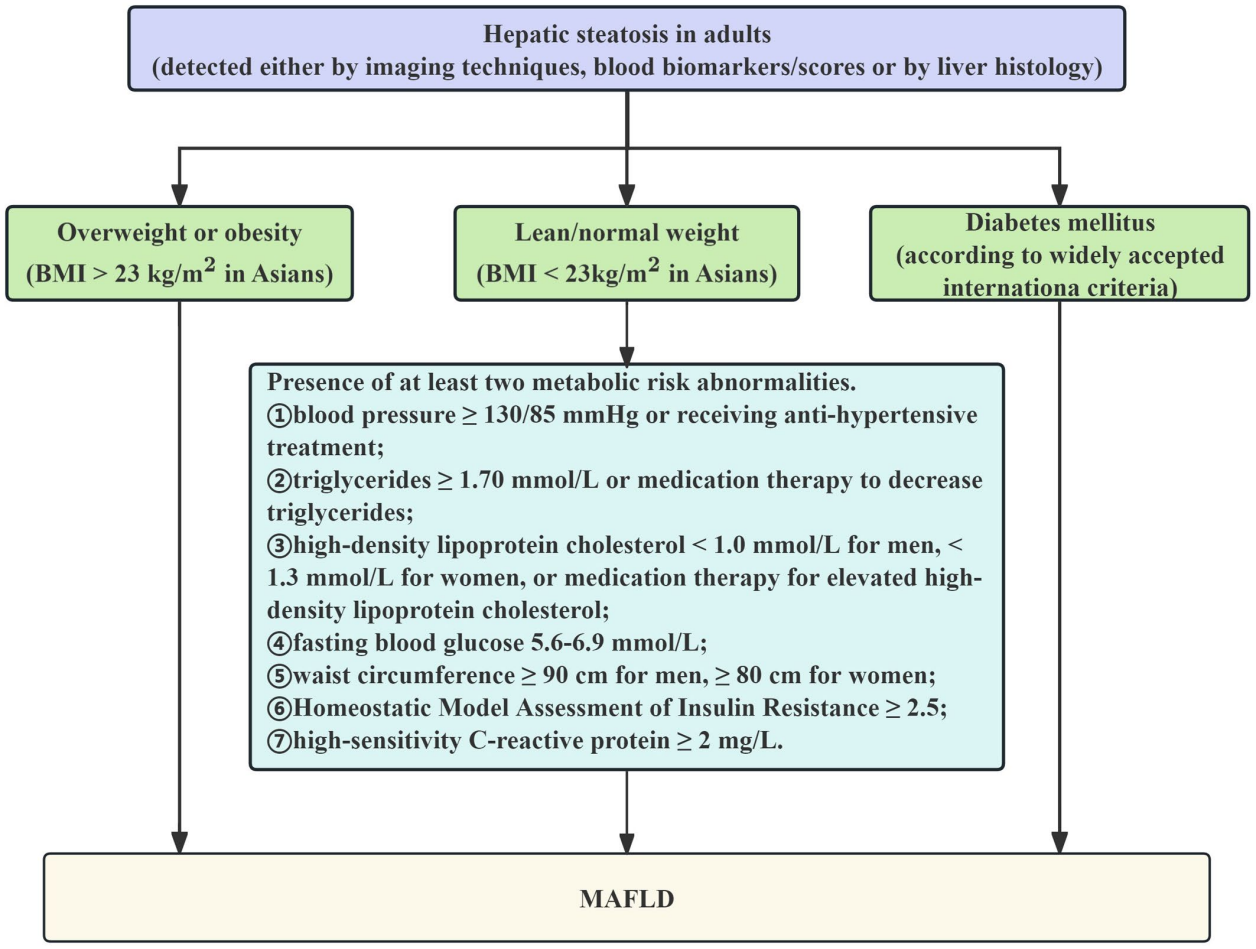
Flowchart of diagnostic criteria for MAFLD.

The global consensus among experts statement on the updated definition of MAFLD, which states that the condition is defined as the presence of fatty liver on abdominal ultrasonography together with one or more of the following criteria, served as the basis for the diagnosis of MAFLD, listed in Figure 1 (28): (1) BMI > 23 kg/m^2^; (2) T2DM; (3)Two or more of the following anomalies related to metabolic risk, including: ① blood pressure ≥ 130/85 mmHg or receiving anti-hypertensive treatment;

② TG ≥ 1.70 mmol/L or medication therapy to decrease TG; ③ HDL-C < 1.0 mmol/L for men, < 1.3 mmol/L for women, or medication therapy for elevated HDL-C; ④ FBG 5.6–6.9 mmol/L; ⑤ waist circumference ≥ 90 cm for men, ≥ 80 cm for women; ⑥ Homeostatic Model Assessment of Insulin Resistance (HOMA-IR) ≥ 2.5; ⑦ high-sensitivity C-reactive protein ≥2 mg/L.

The severity of hepatic steatosis is classified based on liver echogenicity as mild, moderate, and severe (29):

Mild: The liver is of normal size, with a slight diffuse increase in fine echoes; the diaphragm and intrahepatic artery boundaries are clearly visible, and the liver appears brighter than the kidney cortex.

Moderate: The liver size is normal or slightly enlarged, with a moderately distributed rise in fine echoes and a minor impairment in the diaphragm and intrahepatic arteries’ visibility.

Severe: Increased liver volume, which is indicative of a noticeable rise in fine echoes, insufficient penetration of the liver’s posterior right lobe, and little to no visibility of the intrahepatic arteries or diaphragm.

### 2.4 Statistical analysis

Data analysis was carried out employing IBM SPSS Statistics software (version 26.0). The sample size is sufficient (4,663 individuals), hence the central limit theorem states that the sample is thought to follow a normal distribution. According to previous literature (30, 31), the mean ± standard deviations (±SD) was applied to describe continuous variables, and the t-test was employed to evaluate differences between two groups. The analysis of Variance, followed by least significant difference *post-hoc* tests were used for comparing continuous variables among multiple groups. The frequencies (percentages) were applied to describe categorical variables, and the chi-square test was employed to assess differences between two or more groups. Referring to previous cross-sectional studies (32, 33), this research employed univariate and multivariate logistic regression analysis to figure out pertinent risk variables for MAFLD at various degrees of hepatic steatosis severity as determined by ultrasonography. A statistically significant result was defined as a two-tailed *p* value less than 0.05.

## 3 Results

### 3.1 Comparison of general characteristics between MAFLD and non-MAFLD groups

Table 1 compares the general characteristics between MAFLD and non-MAFLD groups. In terms of the category of basic information, the MAFLD group exhibited significantly more indicators of age, female gender, BMI, DBP and SBP than the non-MAFLD group (*p* < 0.05). Regarding the blood routine index category, the MAFLD group had significantly elevated amounts of white blood cell count (WBC) and platelet count (PLT) in contrast to the non-MAFLD group (*p* < 0.05). In regard to glucose and lipid metabolism indices, the MAFLD group exhibited substantially greater levels of TG, low-density lipoprotein cholesterol (LDL-C), total cholesterol (TC), and FBG, instead a lower amount of HDL-C than the non-MAFLD group (*p* < 0.05). In the liver function index category,the MAFLD group demonstrated substantially greater levels of ALT and AST and lower levels of total bilirubin (TBil) relative to the non-MAFLD group (*p* < 0.05). Regarding renal function indices, The MAFLD group demonstrated significantly greater amounts of SUA and significantly less amount of serum creatinine (Scr) and blood urea nitrogen (BUN) contrasted to the non-MAFLD group (*p* < 0.05).

**Table 1:** Comparison of general characteristics between non-MAFLD group and MAFLD group.

Parameters	Non-MAFLD (*n* = 1,678)	MAFLD (*n* = 2,985)	t/χ^2^	*p-*value
Age (years)	71.55 ± 5.09	70.85 ± 4.76	4.634	<0.001
Female [n(%)]	684(40.8%)	1943(65.1%)	258.478	<0.001
BMI (kg/m^2^)	23.65 ± 2.83	27.37 ± 3.09	−41.736	<0.001
SBP (mmHg)	148.23 ± 25.74	154.96 ± 24.92	−8.744	<0.001
DBP (mmHg)	82.84 ± 13.98	87.02 ± 14.41	−9.613	<0.001
WBC (10^9^/L)	5.79 ± 1.56	6.15 ± 1.61	−7.393	<0.001
PLT (10^9^/L)	205.91 ± 55.61	214.44 ± 53.87	−5.130	<0.001
FBG (mmol/L)	6.30 ± 1.72	6.92 ± 2.13	−10.849	<0.001
ALT (U/L)	21.17 ± 12.79	25.26 ± 17.28	−9.226	<0.001
AST (U/L)	24.01 ± 8.37	24.95 ± 14.41	−2.832	0.005
TBil (umol/L)	19.32 ± 7.45	18.25 ± 8.01	4.573	<0.001
TC (mmol/L)	5.31 ± 1.18	5.48 ± 1.24	−4.567	<0.001
TG (mmol/L)	1.31 ± 0.72	1.90 ± 1.35	−19.413	<0.001
HDL-C (mmol/L)	1.96 ± 0.57	1.77 ± 0.48	12.099	<0.001
LDL-C (mmol/L)	3.09 ± 0.84	3.24 ± 0.85	−6.133	<0.001
Scr (umol/L)	75.64 ± 15.60	73.66 ± 19.12	3.624	<0.001
BUN (mmol/L)	6.33 ± 1.79	6.19 ± 1.83	2.704	0.007
SUA (umol/L)	328.21 ± 84.33	344.15 ± 85.65	−6.131	<0.001

Continuous variables used *t* test compare between groups. Categorical variables used chi-square test between groups. MAFLD, metabolic dysfunction-associated fatty liver disease; SBP, systolic blood pressure; BMI, body mass index; PLT, platelet count; DBP, diastolic blood pressure; WBC, white blood cell count; FBG, fasting blood glucose; TBil, total bilirubin; ALT, alanine transaminase; AST, aspartate transaminase; LDL-C, low-density lipoprotein cholesterol; HDL-C, high-density lipoprotein cholesterol; TC, total cholesterol; TG, triglycerides; Scr, serum creatinine; BUN, blood urea nitrogen; SUA, serum uric acid.

### 3.2 Comparison of general characteristics among different grades of hepatic steatosis

As illustrated in Table 2, in the basic information category, the moderate group exhibited considerably greater levels of female gender than the mild group (*p* < 0.05). And the moderate and severe groups had significantly greater DBP value than the mild group (*p* < 0.05). All three groups’ BMI values had a positive correlation with the severity of fatty liver, with statistically significant differences (*p* < 0.05). Conversely, the variations in age and SBP across the three groups were not statistically significant (*p* > 0.05). Regarding the glucose and lipid metabolism index, FBG values demonstrated a positive correlation with the severity of fatty liver in three groups, with significant differences (*p* < 0.05). The moderate and severe groups had significantly greater amounts of TG than the mild group (*p* < 0.05). Nevertheless, LDL-C, HDL-C, and TC values were not substantially different across the groups (*p* > 0.05). In terms of the liver function index, ALT and AST values were positively correlated with fatty liver severity across the groups, with statistically significant differences (*p* < 0.05). Whereas the mild and moderate groups had significantly greater TBil values than the severe group (p < 0.05). Regarding renal function indices, the moderate and severe groups exhibited greater SUA values than the mild group, and these differences were statistically significant (*p* < 0.05). There were no statistically significant variations in Scr and BUN values between the three groups (*p* > 0.05).

**Table 2:** Comparison of general characteristics among different grades of hepatic steatosis in MAFLD.

Parameters	Mild (*n* = 2,104)	Moderate (*n* = 766)	Severe (*n* = 115)	F/χ^2^	*p-*value
Age (years)	70.88 ± 4.79	70.74 ± 4.75	70.96 ± 4.21	0.257	0.773
Female [n(%)]	1,337(63.5%)	527(68.8%)	79(68.7%)	7.504	0.023^a^
BMI (kg/m^2^)	26.90 ± 2.90	28.18 ± 3.03	30.76 ± 3.57	131.083	<0.001^a^^,^^b^^,^^c^
SBP (mmHg)	154.62 ± 25.21	155.62 ± 24.43	156.69 ± 22.82	0.738	0.478
DBP (mmHg)	86.47 ± 14.22	88.19 ± 14.71	89.36 ± 15.32	5.570	0.004^ab^
WBC (10^9^/L)	6.09 ± 1.64	6.24 ± 1.55	6.54 ± 1.46	6.040	0.002^ab^
PLT (10^9^/L)	213.31 ± 52.48	216.56 ± 57.39	221.09 ± 54.47	1.932	0.145
FBG (mmol/L)	6.82 ± 2.09	7.08 ± 2.16	7.69 ± 2.37	12.122	<0.001^a^^,^^b^^,^^c^
ALT (U/L)	24.00 ± 17.32	27.44 ± 16.17	33.90 ± 19.73	26.485	<0.001^a^^,^^b^^,^^c^
AST (U/L)	24.32 ± 15.02	25.96 ± 12.34	29.88 ± 14.56	10.696	<0.001^a^^,^^b^^,^^c^
TBil (umol/L)	18.41 ± 8.58	18.08 ± 6.54	16.62 ± 5.54	2.941	0.043^bc^
TC (mmol/L)	5.45 ± 1.23	5.53 ± 1.24	5.60 ± 1.35	1.856	0.156
TG (mmol/L)	1.82 ± 1.30	2.05 ± 1.46	2.24 ± 1.33	11.779	<0.001^a^^,^^b^
HDL-C (mmol/L)	1.78 ± 0.48	1.75 ± 0.48	1.72 ± 0.45	1.573	0.208
LDL-C (mmol/L)	3.23 ± 0.85	3.30 ± 0.86	3.28 ± 0.88	2.002	0.135
Scr (umol/L)	73.43 ± 16.44	74.20 ± 24.72	74.36 ± 21.95	0.543	0.581
BUN (mmol/L)	6.18 ± 1.66	6.22 ± 2.26	6.11 ± 1.67	0.213	0.809
SUA (umol/L)	339.59 ± 85.54	353.62 ± 83.61	364.37 ± 93.37	10.943	<0.001^a^^,^^b^

Continuous variables used analysis of Variance and least significant difference *post-hoc* tests compare between groups. Categorical variables used chi-square test between groups. MAFLD, metabolic dysfunction-associated fatty liver disease; SBP, systolic blood pressure; BMI, body mass index; PLT, platelet count; DBP, diastolic blood pressure; WBC, white blood cell count; FBG, fasting blood glucose; TBil, total bilirubin; ALT, alanine transaminase; AST, aspartate transaminase; LDL-C, low-density lipoprotein cholesterol; HDL-C, high-density lipoprotein cholesterol; TC, total cholesterol; TG, triglycerides; Scr, serum creatinine; BUN, blood urea nitrogen; SUA, serum uric acid.

a The mild group compared to the moderate group, *p* < 0.0.

b The mild group compared to the severe group, *p* < 0.05.

c The moderate group compared to the severe group, *p* < 0.05.

### 3.3 Analysis of risk factors associated with the severity of hepatic steatosis in MAFLD

**Figure 2:**
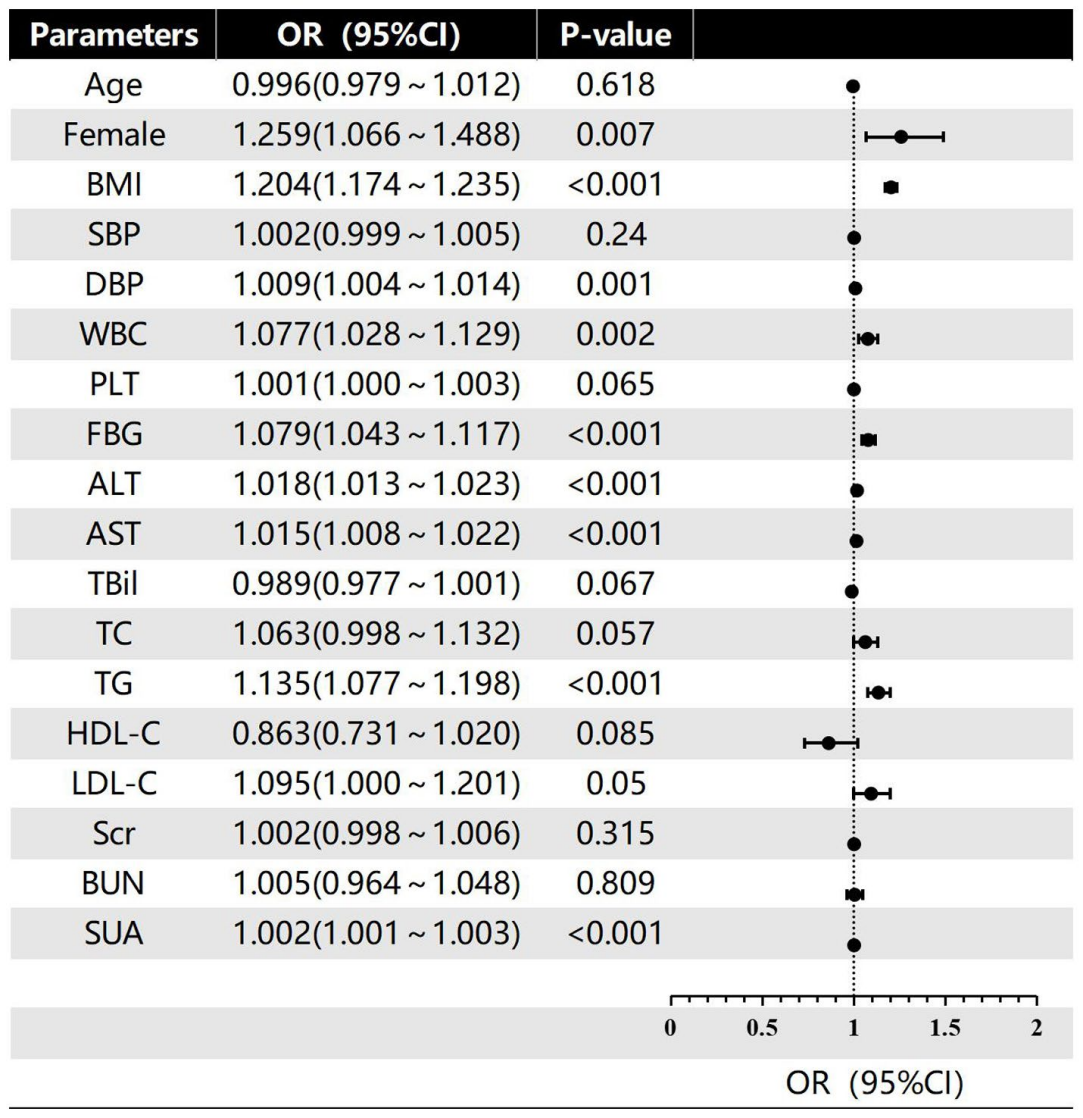
Univariate logistic regression of risk factors for different grades of hepatic steatosis in MAFLD.

**Figure 3:**
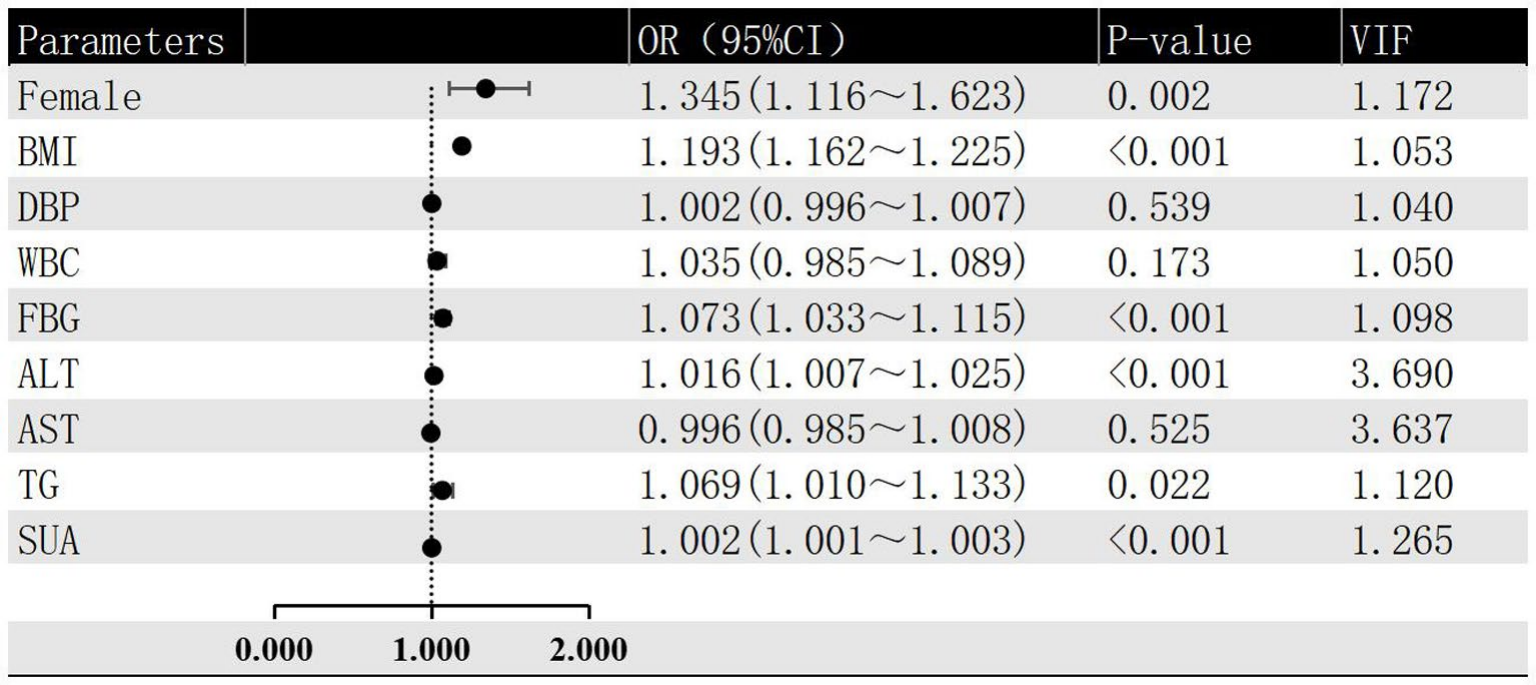
Multivariate logistic regression of risk factors for different grades of hepatic steatosis in MAFLD.

In this investigation, the severity of hepatic steatosis in MAFLD, based on ultrasound grading, was considered as the dependent variable. Univariate logistic regression analysis considered factors such as age, gender, BMI, FBG and blood pressure as independent variables. The results indicated that female gender, BMI, WBC, DBP, ALT, FBG, AST, SUA and TG were all risk factors for the severity of hepatic steatosis in MAFLD (*p* < 0.05; refer to Figure 2). Furthermore, the multivariate logistic regression analysis revealed that female gender, BMI, ALT, FBG, TG, and SUA remained significant after adjusting for all other variables in the model (*p* < 0.05; refer to Figure 3). And female gender (OR 1.345, 95%CI: 1.116 ~ 1.623) and BMI (OR 1.193, 95%CI: 1.162 ~ 1.225) had the greatest effect on hepatic steatosis in MAFLD.

## 4 Discussion

Over the past 4 years, the nomenclature for NAFLD in scientific literature has evolved, introducing terms such as MAFLD and metabolic dysfunction-associated steatotic liver disease (MASLD). Regardless of whether the term used is MAFLD or MASLD, the strong association with metabolic disorders and cardiovascular risk factors has been globally recognized (34). Most patients with MAFLD present with only hepatic steatosis, however, in a subset of patients, the disease may progress to steatohepatitis. A critical event in the pathophysiology of this condition is the immune system is activated. Various factors are associated with the progression of MAFLD, including hypoxia, endoplasmic reticulum stress, insulin resistance, and dyslipidemia, significantly increasing the risk of cirrhosis and hepatocellular carcinoma (35). MAFLD is now diagnosed independently of other liver diseases, allowing clinicians to identify and manage all liver conditions comprehensively within individual patients (36). Community health services play an essential part in identifying and managing MAFLD through clinical assessment and appropriate referrals to specialized care (37). However, in community settings, individuals with advanced fibrosis and cirrhosis caused by MAFLD are frequently disregarded (38). Early diagnosis, particularly in primary healthcare institutions, is essential to address this gap and improve patient oucomes (39).

In our research, values of ALT, FBG, BMI and TG were positively correlated with the severity of hepatic steatosis, consistent with findings by Tutunchi et al. (40). ALT, a key indicator for liver disease diagnosis, effectively reflects the severity of liver damage (41). Serum transaminase values that are mildly to moderately elevated in MAFLD patients may be an indicator of inflammation or liver damage. Liver damage appears to be progressive as there is a positive connection between the developed liver enzyme levels and the degree of hepatic steatosis (42). This might be because MAFLD individuals’ liver cells contain greater concentrations of free fatty acids. Resulting in oxidative stress or increased liver inflammation, which in turn promotes hepatocyte necrosis and degeneration and increases serum ALT levels (43). Additionally, in chronic liver disease, ALT levels are more commonly elevated than AST, except in alcoholic liver disease, where elevated AST levels also indicate hepatocellular injury (44). While AST is often used in MAFLD studies to predict advanced liver fibrosis (45), our multivariable analysis did not indicate a significant role for AST in the progression of steatosis in our cohort.

T2DM has been associated to both liver fibrosis and MAFLD in several reports (46, 47). Severe intrahepatic lipid buildup was strongly correlated with elevated blood triglyceride levels in patients with MAFLD. 188 outpatients were included in a cross-sectional study measuring liver fat content, which found that the primary cause of ApoB elevation was severe intrahepatic lipid accumulation. Based on their connection to cardiovascular diseases and mortality, these anomalies have been demonstrated to be significant indicators for a dismal prognosis (48). Perhaps the explanation for this is that hepatic triglyceride synthesis rises significantly in persons with diabetes, a condition that can be rectified with diabetes remission (49).

A meta-analysis on BMI and MAFLD demonstrated a 3.5-fold increase in MAFLD risk in subjects with higher BMI, indicating a significant dose–response relationship between BMI levels and MAFLD risk (50). Additionally, Cuenza’s research demonstrated an advantageous connection between body mass index and the severity of hepatic steatosis, which is in line with our findings (51). Interestingly, our research demonstrated that people with MAFLD had less TBil values than individuals without MAFLD, and that individuals who had severe hepatic steatosis had less TBil values than subjects with mild and moderate hepatic steatosis. A rise in bilirubin levels has been adversely correlated with the occurrence of MAFLD, while some evidence indicated that low TBil values were linked to a higher risk of MAFLD (52). Exogenous bilirubin supplementation to increase bilirubin levels might be a promising therapeutic strategy for MAFLD (53).

Previous investigations have revealed that MAFLD is particularly prevalent in men, and male gender is regarded as a separate risk indicator for MAFLD (54). However, our study yielded completely different results, likely attributed to our sample consisting solely of elderly women aged 65 and above. The disappearance of gender differences, possibly due to decreased estrogen levels after menopause, could be an important contributing factor to this phenomenon. MAFLD is more common in postmenopausal women, according to epidemiological statistics (55). For instance, in certain clinical studies, the prevalence of MAFLD was significantly greater in postmenopausal women than in men of the same age (19.4% vs. 14.9%), but lower in premenopausal women than in men (12.7% vs. 26%). Postmenopausal women have approximately a 2.4-fold greater likelihood of MAFLD than premenopausal women, revealed to a meta-analysis (56). The decline in estrogen levels leads to hepatic steatosis by reducing fatty acid oxidation and increasing intrahepatic fat synthesis (57). Furthermore, postmenopausal women tend to accumulate more visceral fat compared to premenopausal women, rendering them more susceptible to metabolic disturbances and hepatic steatosis (58). Intestinal flora imbalance, oxidation and antioxidant system imbalance, IR, glucose metabolic problems, and accelerated blood lipids may all be caused by estrogen insufficiency. The advantages of estrogen replacement treatment (ERT), which lowers the prevalence of postmenopausal MAFLD, have been documented in multiple investigations (59). Age has been connected in certain research to an elevated likelihood of MAFLD, encompassing subjects from various age groups (60). However, our study found no significant relationship between age and MAFLD severity in the elderly population, suggesting that age may not be connected to hepatic steatosis severity in this demographic. Van et al.’s prospective cohort analysis indicated that in those 65 years of age and older, fatty liver is not related to all-cause mortality (61).

SUA was determined to be a distinct risk indicator for MAFLD in Tao et al.’s trial (62). This is consistent with our findings. Previous research has indicated that elevated amounts of SUA may cause insulin resistance, which in turn contributes to the accumulation of visceral fat (63). Conversely, lowering SUA levels through hyperuricemia treatment can enhance overall metabolic status and reduce visceral fat deposition (64). Computed tomography studies have independently associated hyperuricemia with hepatic and visceral fat tissue accumulation (65). Liu et al.’s prospective analysis discovered that higher SUA levels significantly increase the risk of MAFLD, with a 17% increase in MAFLD risk for every 60 mg/dL increase in SUA levels (95% CI 9–24%). SUA levels also serve as predictive markers for long-term mortality in MAFLD patients (66).

Our research has an assortment of limitations. Firstly, as a cross-sectional study of elderly MAFLD patients from the Eastern China region, the unique demographic, geographic, and environmental characteristics of this cohort may limit the generalizability of the findings to broader populations or other ethnic groups. Without controlling for potential confounding factors, such as dietary habits, exercise, and smoking, which could impact the accuracy and interpretability of the results. Moreover, the specific genetic predispositions and lifestyle factors prevalent in this region may not be representative of those found in other populations, further restricting the applicability of our results. Therefore, caution should be exercised when applying these findings to other populations, as they may not accurately reflect the distinct characteristics and risk factors present in different regions or ethnic groups. Additionally, the data source from community health service centers might not fully represent the broader elderly population, especially those with poorer health who did not undergo physical examinations. It is necessary to compare the findings with those of other regions or larger-scale studies to comprehensively validate the conclusions. And the cross-sectional design is suitable for identifying associations between variables but cannot establish causal relationships, it is need for future longitudinal studies to confirm causality. Secondly, since fasting serum insulin and C-reactive protein levels were not assessed, both of which are components of metabolic dysregulation that define MAFLD, the diagnosis of MAFLD in certain individuals with normal BMI may be ignored. Lastly, although ultrasound is widely used to detect hepatic steatosis, its sensitivity is lower compared to methods such as MRI or liver biopsy, particularly in cases of mild steatosis. Its limitations include difficulty in accurately quantifying fat infiltration and distinguishing simple steatosis from more severe conditions. This reduced sensitivity may lead to underdiagnosis or misdiagnosis. The diagnostic accuracy of ultrasound significantly depends on the operator’s expertise and experience, potentially leading to variability in results. There is no universally accepted standardization for ultrasound-based diagnostic criteria for severity of fatty liver, which may result in inconsistent diagnoses across studies and clinical practices.

In conclusion, this research examined the metabolic profiles of elderly MAFLD patients with varying severity of hepatic steatosis as determined by ultrasound. The severity of hepatic steatosis in elderly MAFLD patients was significantly connected with female gender, BMI, FBG, ALT, TG and SUA levels. Since diet and exercise are the cornerstones of therapy for MAFLD, no particular drugs are currently licensed for its treatment. Therefore, early diagnosis and prevention are essential, and the aforementioned indicators can serve as valuable references for early MAFLD screening and diagnosis.

## Data Availability

The original contributions presented in the study are included in the article/supplementary material, further inquiries can be directed to the corresponding author.
